# Supplementary material for: Multi‐dataset identification of innovative feature genes and molecular mechanisms in keratoconus
Source: J Cell Mol Med. 2024 Sep 19;28(18):e70079. doi: 10.1111/jcmm.70079 (PMC11412914; doi:10.1111/jcmm.70079)
Supplement: Supplementary file 2 — Material S2. [file JCMM-28-e70079-s002.zip › 7.GSEA analysis/1.ARL11/KEGG_TIGHT_JUNCTION.html]

Details for gene set KEGG\_TIGHT\_JUNCTION[GSEA]

|  || Dataset | ARL11.group.cls#h\_versus\_l |
| Phenotype | group.cls#h\_versus\_l |
| Upregulated in class | l |
| GeneSet | KEGG\_TIGHT\_JUNCTION |
| Enrichment Score (ES) | -0.29223454 |
| Normalized Enrichment Score (NES) | -1.0890739 |
| Nominal p-value | 0.32364342 |
| FDR q-value | 1.0 |
| FWER p-Value | 0.997 |
Table: GSEA Results Summary

  

Fig 1: Enrichment plot: KEGG\_TIGHT\_JUNCTION      
 Profile of the Running ES Score & Positions of GeneSet Members on the Rank Ordered List

  

| SYMBOL | TITLE | RANK IN GENE LIST | RANK METRIC SCORE | RUNNING ES | CORE ENRICHMENT || 1 | CLDN5 | na | 852 | 0.409 | 0.0084 | No |
| 2 | RAB3B | na | 2694 | 0.255 | -0.0242 | No |
| 3 | PRKCQ | na | 2774 | 0.251 | -0.0062 | No |
| 4 | CLDN15 | na | 4053 | 0.199 | -0.0270 | No |
| 5 | CLDN10 | na | 4680 | 0.176 | -0.0309 | No |
| 6 | MYH15 | na | 4981 | 0.167 | -0.0261 | No |
| 7 | CLDN18 | na | 5112 | 0.162 | -0.0168 | No |
| 8 | CLDN9 | na | 5240 | 0.159 | -0.0076 | No |
| 9 | EPB41 | na | 5663 | 0.146 | -0.0080 | No |
| 10 | CLDN22 | na | 5685 | 0.145 | 0.0031 | No |
| 11 | CLDN20 | na | 6192 | 0.132 | -0.0008 | No |
| 12 | CLDN3 | na | 6601 | 0.122 | -0.0028 | No |
| 13 | CLDN23 | na | 6669 | 0.120 | 0.0049 | No |
| 14 | MYH4 | na | 6714 | 0.118 | 0.0132 | No |
| 15 | MYH13 | na | 6896 | 0.114 | 0.0172 | No |
| 16 | CLDN19 | na | 7118 | 0.109 | 0.0196 | No |
| 17 | ASH1L | na | 7594 | 0.100 | 0.0139 | No |
| 18 | GNAI3 | na | 7891 | 0.095 | 0.0130 | No |
| 19 | EPB41L1 | na | 8416 | 0.084 | 0.0047 | No |
| 20 | MYH11 | na | 8489 | 0.083 | 0.0093 | No |
| 21 | MYH6 | na | 8587 | 0.081 | 0.0131 | No |
| 22 | EPB41L2 | na | 9061 | 0.074 | 0.0054 | No |
| 23 | PARD6G | na | 9225 | 0.071 | 0.0064 | No |
| 24 | MYL5 | na | 9267 | 0.071 | 0.0110 | No |
| 25 | AKT2 | na | 9348 | 0.070 | 0.0143 | No |
| 26 | SYMPK | na | 9381 | 0.069 | 0.0189 | No |
| 27 | AMOTL1 | na | 9703 | 0.064 | 0.0148 | No |
| 28 | MYH7B | na | 10179 | 0.058 | 0.0058 | No |
| 29 | CLDN7 | na | 10343 | 0.056 | 0.0056 | No |
| 30 | PRKCA | na | 10617 | 0.052 | 0.0019 | No |
| 31 | KRAS | na | 10630 | 0.052 | 0.0058 | No |
| 32 | MYH14 | na | 10974 | 0.048 | -0.0003 | No |
| 33 | PPP2R2B | na | 10976 | 0.048 | 0.0035 | No |
| 34 | EXOC3 | na | 11000 | 0.047 | 0.0067 | No |
| 35 | CTTN | na | 11588 | 0.040 | -0.0070 | No |
| 36 | LLGL2 | na | 11604 | 0.040 | -0.0042 | No |
| 37 | PPP2R2D | na | 11894 | 0.037 | -0.0095 | No |
| 38 | MYH2 | na | 11936 | 0.037 | -0.0077 | No |
| 39 | MYH9 | na | 12105 | 0.035 | -0.0097 | No |
| 40 | EPB41L3 | na | 12201 | 0.034 | -0.0097 | No |
| 41 | CSNK2A1 | na | 12274 | 0.034 | -0.0091 | No |
| 42 | SPTAN1 | na | 12393 | 0.032 | -0.0099 | No |
| 43 | MYH8 | na | 12614 | 0.030 | -0.0138 | No |
| 44 | ACTB | na | 12961 | 0.027 | -0.0216 | No |
| 45 | GNAI2 | na | 13045 | 0.027 | -0.0218 | No |
| 46 | CGN | na | 13186 | 0.025 | -0.0238 | No |
| 47 | ACTN4 | na | 13347 | 0.024 | -0.0265 | No |
| 48 | YBX3 | na | 13354 | 0.024 | -0.0247 | No |
| 49 | LLGL1 | na | 13379 | 0.024 | -0.0235 | No |
| 50 | MAGI3 | na | 13440 | 0.023 | -0.0233 | No |
| 51 | ACTN1 | na | 13743 | 0.021 | -0.0304 | No |
| 52 | PTEN | na | 14202 | 0.017 | -0.0422 | No |
| 53 | CTNNB1 | na | 14653 | 0.014 | -0.0541 | No |
| 54 | PRKCZ | na | 14777 | 0.013 | -0.0566 | No |
| 55 | EXOC4 | na | 14946 | 0.011 | -0.0606 | No |
| 56 | PPP2R2C | na | 14948 | 0.011 | -0.0597 | No |
| 57 | CTNNA1 | na | 15057 | 0.011 | -0.0619 | No |
| 58 | RRAS | na | 15323 | 0.009 | -0.0688 | No |
| 59 | MYH10 | na | 15785 | 0.005 | -0.0817 | No |
| 60 | TJP1 | na | 15790 | 0.005 | -0.0814 | No |
| 61 | CLDN4 | na | 15840 | 0.005 | -0.0825 | No |
| 62 | PRKCI | na | 16036 | 0.003 | -0.0878 | No |
| 63 | MYL12B | na | 16348 | 0.001 | -0.0967 | No |
| 64 | PRKCD | na | 16865 | -0.002 | -0.1114 | No |
| 65 | MYH7 | na | 16998 | -0.003 | -0.1150 | No |
| 66 | PRKCG | na | 17545 | -0.007 | -0.1302 | No |
| 67 | AKT1 | na | 18085 | -0.011 | -0.1449 | No |
| 68 | CSNK2B | na | 18138 | -0.011 | -0.1455 | No |
| 69 | CDC42 | na | 18361 | -0.013 | -0.1509 | No |
| 70 | HRAS | na | 18435 | -0.013 | -0.1519 | No |
| 71 | F11R | na | 18558 | -0.014 | -0.1543 | No |
| 72 | CDK4 | na | 18688 | -0.015 | -0.1568 | No |
| 73 | MYH3 | na | 18992 | -0.017 | -0.1642 | No |
| 74 | CLDN8 | na | 19025 | -0.017 | -0.1638 | No |
| 75 | MYL12A | na | 19058 | -0.017 | -0.1633 | No |
| 76 | TJP2 | na | 19172 | -0.018 | -0.1651 | No |
| 77 | ACTN2 | na | 19694 | -0.022 | -0.1784 | No |
| 78 | OCLN | na | 19863 | -0.023 | -0.1814 | No |
| 79 | PPP2R1A | na | 19944 | -0.023 | -0.1818 | No |
| 80 | CSNK2A2 | na | 20099 | -0.024 | -0.1843 | No |
| 81 | ACTG1 | na | 20219 | -0.025 | -0.1857 | No |
| 82 | TJAP1 | na | 20968 | -0.031 | -0.2048 | No |
| 83 | PRKCH | na | 21082 | -0.032 | -0.2055 | No |
| 84 | PARD3 | na | 21300 | -0.034 | -0.2091 | No |
| 85 | RAB13 | na | 21829 | -0.038 | -0.2213 | No |
| 86 | RHOA | na | 21886 | -0.038 | -0.2198 | No |
| 87 | VAPA | na | 23278 | -0.053 | -0.2557 | No |
| 88 | CRB3 | na | 23399 | -0.055 | -0.2548 | No |
| 89 | TJP3 | na | 23515 | -0.056 | -0.2536 | No |
| 90 | HCLS1 | na | 23900 | -0.061 | -0.2598 | No |
| 91 | CASK | na | 23988 | -0.062 | -0.2573 | No |
| 92 | SRC | na | 24048 | -0.063 | -0.2539 | No |
| 93 | CLDN16 | na | 24265 | -0.066 | -0.2548 | No |
| 94 | CLDN6 | na | 24385 | -0.068 | -0.2528 | No |
| 95 | MYL9 | na | 24535 | -0.070 | -0.2515 | No |
| 96 | YES1 | na | 24999 | -0.076 | -0.2587 | No |
| 97 | RRAS2 | na | 25031 | -0.076 | -0.2534 | No |
| 98 | PPP2CA | na | 25060 | -0.077 | -0.2480 | No |
| 99 | PARD6A | na | 25242 | -0.079 | -0.2469 | No |
| 100 | MPDZ | na | 25950 | -0.090 | -0.2601 | No |
| 101 | MAGI2 | na | 26002 | -0.090 | -0.2543 | No |
| 102 | CLDN1 | na | 26061 | -0.091 | -0.2486 | No |
| 103 | PPP2R2A | na | 26307 | -0.095 | -0.2480 | No |
| 104 | CTNNA2 | na | 26542 | -0.099 | -0.2467 | No |
| 105 | PPP2R1B | na | 26783 | -0.104 | -0.2453 | No |
| 106 | GNAI1 | na | 27299 | -0.115 | -0.2509 | No |
| 107 | PPP2CB | na | 27315 | -0.115 | -0.2420 | No |
| 108 | AKT3 | na | 27547 | -0.120 | -0.2390 | No |
| 109 | NRAS | na | 27790 | -0.126 | -0.2359 | No |
| 110 | MYL7 | na | 29740 | -0.180 | -0.2777 | Yes |
| 111 | MAGI1 | na | 29920 | -0.185 | -0.2679 | Yes |
| 112 | IGSF5 | na | 30323 | -0.199 | -0.2635 | Yes |
| 113 | MYL2 | na | 30743 | -0.212 | -0.2585 | Yes |
| 114 | PARD6B | na | 31169 | -0.227 | -0.2524 | Yes |
| 115 | CLDN11 | na | 31438 | -0.237 | -0.2411 | Yes |
| 116 | MRAS | na | 31743 | -0.250 | -0.2297 | Yes |
| 117 | PRKCE | na | 32224 | -0.270 | -0.2217 | Yes |
| 118 | CLDN14 | na | 32448 | -0.283 | -0.2053 | Yes |
| 119 | MYH1 | na | 32538 | -0.290 | -0.1844 | Yes |
| 120 | CTNNA3 | na | 32684 | -0.300 | -0.1644 | Yes |
| 121 | CLDN17 | na | 33095 | -0.329 | -0.1497 | Yes |
| 122 | JAM2 | na | 33406 | -0.352 | -0.1303 | Yes |
| 123 | CLDN2 | na | 33555 | -0.365 | -0.1051 | Yes |
| 124 | PRKCB | na | 33863 | -0.399 | -0.0818 | Yes |
| 125 | MYL10 | na | 34046 | -0.426 | -0.0526 | Yes |
| 126 | JAM3 | na | 34122 | -0.440 | -0.0193 | Yes |
| 127 | ACTN3 | na | 34217 | -0.455 | 0.0148 | Yes |
Table: GSEA details [plain text format]

  

Fig 2: KEGG\_TIGHT\_JUNCTION      
 Blue-Pink O' Gram in the Space of the Analyzed GeneSet

  

Fig 3: KEGG\_TIGHT\_JUNCTION: Random ES distribution      
 Gene set null distribution of ES for **KEGG\_TIGHT\_JUNCTION**
